# Incorporating Scale Uncertainty into Differential Expression Analyses Using ALDEx2

**DOI:** 10.1002/cpz1.70307

**Published:** 2026-02-04

**Authors:** Scott J. Dos Santos, Gregory B. Gloor

**Affiliations:** ^1^ Department of Biochemistry, Schulich School of Medicine and Dentistry Western University Ontario Canada

**Keywords:** ALDEx2, differential abundance, differential expression, metagenomics, RNA‐seq

## Abstract

Differential abundance or expression analyses are routinely performed on metagenomic, metatranscriptomic, and amplicon sequencing data. In such datasets, analysts usually have no information regarding the true scale (i.e., size) of the microbial community or sample under study, with inter‐sample differences in sequencing depth instead being driven by technical variation rather than biological factors. Recent work has demonstrated that normalizations used in all analysis tools make incorrect assumptions about the biological scale of the system in question, leading to unacceptably high false‐discovery rates in the output. To mitigate this, analysts can acknowledge and account for the uncertainty of the overall system scale during normalization by building scale models of the data—a feature that has been integrated into the ALDEx2 R package. Here, we provide reproducible examples that demonstrate how to incorporate scale models into differential expression analyses of RNA‐seq data using bulk transcriptome and metatranscriptomic datasets, as well as the consequences of not doing so. We also show how to use the output of ALDEx2 to create high‐level exploratory visualizations of their data through principal component analysis. © 2026 The Author(s). *Current Protocols* published by Wiley Periodicals LLC.

**Basic Protocol 1**: Using a simple scale model for differential expression analysis to avoid dual‐cutoff *P* value/significance thresholds

**Basic Protocol 2**: Implementing a full informed scale model to correct scale‐related data asymmetry in differential expression analyses

**Basic Protocol 3**: Visualizing ALDEx2 outputs using a compositional approach: Principal component analysis

## Introduction

High‐throughput sequencing (HTS) is a popular tool for investigating differences in composition and/or gene expression between microbial communities or cell populations. Commonly used approaches include bulk, single‐cell, or meta transcriptome profiling by RNA‐seq for gene expression analysis; amplicon sequencing of universal barcoding genes; and shotgun metagenomic sequencing (Manzoni et al., 2016). At each sampling step of the laboratory work, from the initial DNA/RNA extraction to library construction/multiplexing and loading onto a flow cell, critical information regarding the scale of the community is lost (e.g., total number of nucleic acid fragments, total gene expression levels, or overall microbial load). The final output from a sequencing experiment is usually a table of counts of genes or other features across multiple samples. These counts represent a small subset of the final DNA library loaded onto the sequencer, which is itself a subset of the individually prepared sequencing libraries, which is itself a subset of the original DNA extract, and so on, all the way back to the sample taken from the original community. Importantly, the instrument also imposes an upper limit on the number of molecules that can be sequenced (Gloor et al., 2017). Therefore, information contained in the sequencing output only informs us of the relative abundances of the genes/features. Scale information can be obtained by complementary approaches alongside sequencing, including estimating total bacterial loads via qPCR or droplet digital (dd)PCR of the 16S rRNA gene (Barlow et al., 2020); spiking in a known number of intact cells or molecules that can be used to back‐normalize relative abundance values (Rao et al., 2021); quantifying the total number of bacterial cells using flow cytometry (Lin et al., 2019); and estimating the number of viable cells through microbial cultivation and CFU/ml determination (Rolling et al., 2021). These approaches are not routinely employed in most microbiome studies, however.

Analysis of HTS data is inherently variable due to the lack of information about the absolute size or “scale”. Notably, the false‐discovery rate (FDR) is often poorly controlled by many popular tools and inference of differentially expressed genes can be subjective because it is routinely based upon non‐standard cutoffs (e.g., fold‐change) (Ebrahimpoor & Goeman, 2021; Gloor et al., 2025; Li et al., 2022). Such approaches have been shown to inadvertently inflate the rate of false discoveries (Bourgon et al., 2010; Ebrahimpoor & Goeman, 2021). Likewise, many sequencing datasets reflect biological situations where there is an innate difference in the overall mean difference between the conditions of interest or where there may be a prevalence difference that manifests as a sparse data table where sets of genes are present in samples from one condition but not the other (Nixon et al., 2024). Multiple tools perform poorly on such datasets (Gloor et al., 2025; Li et al., 2022). Often in HTS analyses, scale itself is a major confounder of differential expression/abundance, and recognition of this in the field of transcriptomics drove the development of normalization methods (Bullard et al., 2010). Examples of scale issues that complicate HTS analyses pervade the literature: total and mRNA levels are increased approximately 2‐fold and 3.5‐fold, respectively, in B lymphocytes overexpressing the oncogene c‐Myc compared to non‐transformed cells (Nie et al., 2012). However, follow‐up investigations by the same group determined that this could drastically impact transcriptome analysis unless a spike‐in normalization was performed (Lovén et al., 2012). Similarly, total bacterial loads within the vaginal microbiome differ between states of health and bacterial vaginosis by approximately 10‐ to 100‐fold (Zozaya‐Hinchliffe et al., 2010). Failing to account for this leads to many false‐positive and false‐negative findings during differential expression analysis (Dos Santos et al., 2024; Gloor et al., 2025). Finally, differing growth rates and RNA contents have been observed between wild‐type and mutant strains of bacteria, fungi, and eukaryotic cell lines, which, as above, hinders one's ability to infer truly differential genes from scale‐related artefactual findings (Lin & Amir, 2018; Scott et al., 2010; Yoshikawa et al., 2011).

When conceptualizing the scale problem in HTS data, Nixon and colleagues demonstrated that all tools used for differential expression/abundance analysis make varying assumptions regarding scale during normalization and that these assumptions are demonstrably wrong (Nixon et al., 2024). This is one major reason that different normalizations often return vastly different results when applied to the same input data. The original ALDEx2 model is guilty of the same: an inherent and incorrect assumption is made relating the geometric mean of the compositional data to the system scale (Fernandes et al., 2014; Nixon et al., 2024). To address this shortcoming, Nixon et al. used a series of simulations and algebraic proofs to show that modifying this assumption by introducing uncertainty around the scale (i.e., by modeling) could greatly improve both FDR control and reproducibility of the results. ALDEx2 builds a Bayesian posterior model of the HTS count data using Monte Carlo sampling and estimates the underlying technical variation (i.e., what one would see if the same experiment was repeated many times) (Fernandes et al., 2014). These imputed data are subject to a centered log‐ratio (CLR) transformation before testing the null hypothesis that mean log‐ratio differences between groups are not different, with the results averaged across the Monte Carlo instances. Modeling of scale uncertainty was incorporated into the log‐ratio transformation step, and this explicitly accounts for the fact that any individual normalization is wrong but the uncertainty provides an estimate of many reasonable normalizations. The exact details of scale modeling in ALDEx2 are discussed elsewhere (Gloor et al., 2025; McGovern et al., 2023; Nixon et al., 2024).

Here, we describe the steps used to perform differential expression analyses on several example datasets using the scale‐aware ALDEx2 package, which can be applied to any other HTS dataset. We use a highly replicated yeast transcriptome *Snf2* knockout dataset to show how “simple” scale models can be used to eliminate the need for dual *p*‐value and fold‐change cutoffs, which are commonly used to limit lists of differentially expressed genes down to a manageable number of genes likely to be truly differential. Further, we use a vaginal metatranscriptome dataset that represents a worst‐case scenario (where there are vast differences in scale and gene content between conditions) to show how a more complex full‐scale model can be constructed to overcome this issue. Finally, we demonstrate how to determine the effect of increasing amounts of scale uncertainty on a given dataset and provide insight on how to select an appropriate value.

## Basic Protocol 1 USING A SIMPLE SCALE MODEL FOR DIFFERENTIAL EXPRESSION ANALYSIS TO AVOID DUAL‐CUTOFF *P* VALUE/SIGNIFICANCE THRESHOLDS

In a highly replicated yeast transcriptome dataset generated by Gierliński et al. (2015), a majority of genes (65‐80%) were identified as differential by commonly used, scale‐naïve tools such as DESeq2, edgeR, and ALDEx2 (using the original algorithm as in Fernandes et al., 2014). This dataset comprises 48 lab (growth technical) replicates of wild‐type *Saccharomyces cerevisiae* versus an *Snf* *2* knockout strain. In a benchmark study of differential expression tools using the same data, the authors recommended employing a fold‐change threshold to the list of significantly differentially expressed genes to control for potential false discoveries (i.e., genes with a low/marginal fold change between conditions with *P* < 0.05). There are no universally agreed upon values for either threshold and therefore different researchers are free to choose their own *P* value or log‐fold‐change cutoffs, often using volcano plots, which are known to inflate the FDR further (Ebrahimpoor & Goeman, 2021). In this protocol, we provide an example of how adding a small amount of scale uncertainty to the ALDEx2 model can achieve a similar result of controlling for false‐positive findings without the need for arbitrary thresholds.

### Materials

Feature table containing the number of reads per feature (i.e., gene/taxon) across every sample in the dataset. Here we use data from Gierliński et al. (2015) (see data/yeast_counts.txt from the current study's GitHub repository). Samples should be in columns; features in rows.
Metadata table containing group membership information. Again, we use data from Gierliński et al. (2015) (see data/yeast_metadata.txt from the current study's GitHub repository).
Computer with R and RStudio installed and >8 GB RAM. ALDEx2 and its dependencies must be installed from Bioconductor (pre‐computed ALDEx2 outputs are provided for replication of this protocol as an alternative for those who do not meet this requirement). For this protocol, R (v4.5.1) and RStudio (2024.12.1, build 563) were used, along with the following R packages: BiocManager (v1.30.26), ALDEx2 (v1.41), dplyr (v1.1.4), ggplot2 (v3.5.2), and patchwork (v1.3.1).

#### Environment setup

1 Install the ALDEx2 package from Bioconductor. This need only be performed once.


if (!require(“BiocManager”, quietly = TRUE))
install.packages(“BiocManager”)
BiocManager::install(“ALDEx2”)

2 Load the ALDEx2 package and then load the count data and metadata tables directly from GitHub.


library(ALDEx2)
url.gene <‐ “https://github.com/scottdossantos/currprotSDS/raw/refs/heads/main/data/yeast_counts.txt”
yst.gene <‐ read.table(url.gene, sep = “\t”, header = T, quote = “”, row.names = 1)
url.meta <‐ “https://github.com/scottdossantos/currprotSDS/raw/refs/heads/main/data/yeast_metadata.txt”
yst.meta <‐ read.table(url.meta, sep = “\t”, header = T, quote = “”, row.names = 1)

#### Differential expression analysis with ALDEx2

3 Set the seed value (required for exact replication of results on GitHub) and run the centered log‐ratio transformation with half a standard deviation of scale uncertainty, specifying group membership, the type of denominator to use in the geometric mean calculation (all features), and the number of Monte Carlo instances to generate.


set.seed(2025)
yst.5.clr <‐ aldex.clr(reads = yst.gene, conds = yst.meta$group, mc.samples = 128, denom = “all”, gamma = 0.5, verbose = TRUE)

  For further information on the gamma parameter, see Critical Parameters.
4 For each feature, calculate the median dispersion of log‐ratio abundances within groups and the median difference in log‐ratio abundances between groups. This is performed for all 128 Monte Carlo instances and a median of medians is returned.


yst.5.clr.e <‐ aldex.effect(clr = yst.5.clr, verbose = TRUE,include.sample.summary = TRUE)

  When include.sample.summary is set to true, the output of aldex.effect will return the median log‐ratio abundance values of each feature per sample (i.e., averaged across all 128 Monte Carlo instances), which can be used to plot a compositional biplot following principal component analysis.
5 Conduct parametric and nonparametric tests to identify features for which the mean difference in the log‐ratio abundance between groups is significantly different, adjusting for multiple testing using the Benjamini‐Hochberg correction.


yst.5.clr.t <‐ aldex.ttest(clr = yst.5.clr, verbose = TRUE)

  For applicable study designs, it is possible to specify paired t‐tests by adding the call paired.test = TRUE.

#### Visualization of differential expression analysis output

**Figure 1:**
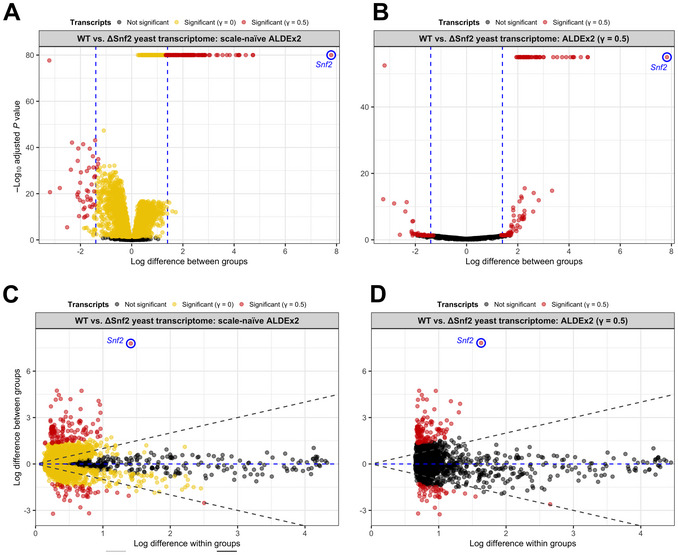
Using a simple scale model eliminates the need for a dual *P*‐value/fold‐change threshold. (**A,B**) Count data from the yeast transcriptome dataset underwent log‐ratio transformation using the ALDEx2 package with (**A**) and without (**B**) modeling scale uncertainty (gamma = 0.5). (**C,D**) Effect sizes and posterior predictive *P* values were calculated for all features, comparing wild‐type and *Snf2* knockout cells.

6 Visualize the output of ALDEx2 by creating volcano plots (difference in log‐ratio abundance between groups vs. –log_10_ *P* values) and effect plots (difference in log‐ratio abundances between groups vs. dispersion of log‐ratio) for this analysis. ALDEx2 has a built‐in function for creating basic versions of these plots (not shown), but we also show the same data plotted using the ggplot2 R package in Figure 1 (see GitHub for ggplot2 plotting code).

yst.5.clr.all <‐ rbind(yst.5.clr.e, yst.5.clr.t)
aldex.plot(yst.5.clr.all, type = “volcano”)
aldex.plot(yst.5.clr.all, type = “MW”)

## Basic Protocol 2 IMPLEMENTING A FULL INFORMED SCALE MODEL TO CORRECT SCALE‐RELATED DATA ASYMMETRY IN DIFFERENTIAL EXPRESSION ANALYSES

In a study of vaginal metatranscriptomes from healthy vs. bacterial vaginosis (BV) patients, we showed how this data represents a worst‐case scenario of scale‐related problems in omics data analysis (Dos Santos et al., 2024). This dataset consists of 42 samples from two separate studies: 20 from healthy or BV patients from Canada (Macklaim & Gloor, 2018) and 22 from BV patients from Germany pre‐ and post‐successful treatment with metronidazole (Deng et al., 2018). Analysts must contend with (1) differing total bacterial loads of approximately two orders of magnitude (Zozaya‐Hinchliffe et al., 2010) and (2) vastly different genomic content in the metatranscriptomes between healthy and BV patients (France et al., 2022). These compositional and scale differences make it challenging to determine differentially expressed genes within this dataset. It is possible to reduce the impact of these scale‐related issues by analyzing this data one organism at a time; however, this precludes system‐wide analyses that underpin the rationale of using metaomics approaches. When these data were analyzed as an entire system, many housekeeping functions were present within the list of differentially expressed genes, which is unintuitive given our understanding of molecular biology and the routine use of such genes as invariant reference sequences or proteins in qPCR or western blotting experiments (Joshi et al., 2022). In this protocol, we show how analysts can implement an informed scale model of their data with ALDEx2, using housekeeping functions to inform on the scale difference between groups.

### Materials

Feature table containing the number of reads per feature (i.e., gene/taxon) across every sample in the dataset. Here we use data from Dos Santos et al. (2024) (see data/mts_counts.txt from this study's GitHub repository). Samples should be in columns; features in rows.
Metadata table containing group membership information. Again, we use data from Dos Santos et al. (2024) (see data/ mts_metadata.txt from the current study's GitHub repository).
Means of identifying the functions of each gene or transcript contained in the feature table. Here we use KEGG orthology terms and their corresponding pathways provided by the authors of the non‐redundant VIRGO database of vaginal microorganisms (see data/mts_virgo_KOs.txt and data/mts_KOlookup.txt from the current study's GitHub repository).
Computer with R and RStudio installed and >8 GB RAM (ore‐computed ALDEx2 outputs are provided for replication of this protocol as an alternative for those who do not meet this requirement). For this protocol, R (v4.5.1) and RStudio (2024.12.1, build 563) were used, along with the following R packages: BiocManager (v1.30.26), ALDEx2 (v1.41), CoDaSeq (v0.99.7), dplyr (v1.1.4), ggplot2 (v3.5.2), and patchwork (v1.3.1).

#### Environment setup

1 Install the ALDEx2 package from Bioconductor. This need only be performed one time.


if (!require(“BiocManager”, quietly = TRUE))
install.packages(“BiocManager”)
BiocManager::install(“ALDEx2”)

2 Load the required libraries, gene count table, metadata, and functional lookup tables directly from GitHub.


library(ALDEx2)
url.gene <‐ “https://github.com/scottdossantos/currprotSDS/raw/refs/heads/main/data/mts_counts.txt”
mts.gene <‐ read.table(url.gene, sep = “\t”, header = T, quote = “”, row.names = 1)
url.meta <‐ “https://github.com/scottdossantos/currprotSDS/raw/refs/heads/main/data/mts_metadata.txt”
mts.meta <‐ read.table(url.meta, sep = “\t”, header = T, quote = “”, row.names = 1)
url.func <‐ “https://github.com/scottdossantos/currprotSDS/raw/refs/heads/main/data/mts_KOlookup.txt”
mts.func <‐ read.table(url.func, sep = “\t”, header = T, quote = “”, row.names = 1)
url.virg <‐ “https://github.com/scottdossantos/currprotSDS/raw/refs/heads/main/data/mts_virgo_KOs.txt”
mts.virg <‐ read.table(url.virg, sep = “\t”, header = F, quote = “”, row.names = 1)

#### Aggregate gene count data by function

3 Match the gene IDs to the corresponding functions in the gene vs. KEGG orthology term lookup table.


ko.virgo <‐ mts.virg[[1]][match(rownames(mts.gene), rownames(mts.virg))]

4 For each KEGG orthology term in the dataset, sum all read counts for every gene in the dataset assigned to a given orthology term. Clean up this dataframe after aggregating.


mts.ko <‐ aggregate(mts.gene, by = list(ko.virgo), FUN = sum)
rownames(mts.ko) <‐ mts.ko$Group.1
mts.ko$Group.1 <‐ NULL

  This example uses KEGG orthology terms, but you may use whichever functional classification system you prefer as long as you have a way of identifying functions in your system that you know (or have reason to believe) are invariant between groups.

#### Determine background scale values per group

5 Set the seed value (required for exact replication of results on GitHub), then run a scale‐naïve ALDEx2 centered log‐ratio transformation on the data using an incredibly small (i.e., virtually no) scale uncertainty.


set.seed(2025)
scale.0.clr <‐ aldex.clr(reads = mts.ko, conds = mts.meta$group, mc.samples = 128, denom = “all”, gamma = 1e‐3, verbose = TRUE)

  For further information on the gamma parameter, see Critical Parameters.
6 Isolate the scale estimates per Monte Carlo instance from the log‐ratio transformation output and calculate the mean background scale values.


bg.scale.0 <‐ scale.0.clr@scaleSamps
bg.scale.h.0 <‐ mean(rowMeans(bg.scale.0)[which(mts.meta$group == “Healthy”)])
bg.scale.bv.0 <‐mean(rowMeans(bg.scale.0)[which(mts.meta$group == “BV”)])
abs(bg.scale.h.0 – bg.scale.bv.0)
2^abs(bg.scale.h.0 – bg.scale.bv.0)

  The @scaleSamps element of the log‐ratio transformation output contains all scale estimates used for each sample across all Monte Carlo instances; the mean of the rows for all samples belonging to a given condition corresponds to the background scale values for each sample. For this example, there is a log_2_ difference in scale between groups of 3.005442, equating to a roughly 8‐fold overall difference in scale between samples from healthy patients vs. BV patients.
7 Subset the functional feature table (i.e., table of counts per KEGG orthology term across all samples) to extract only those related to housekeeping functions. The explicit assumption being made here is that housekeeping functions should be roughly at the same scale.


hk.func <‐ which(mts.func$pathway %in% c(“Ribosome”,“Glycolysis / Gluconeogenesis”,“Aminoacyl‐tRNA biosynthesis”))
mts.ko.hk <‐ mts.ko[hk.func,]

8 Run a centered log‐ratio transformation on these housekeeping functions using the same parameters as the full dataset.


set.seed(2025)
hk.clr <‐ aldex.clr(reads = mts.ko.hk, conds = mts.meta$group, mc.samples = 128, denom = “all”, gamma = 1e‐3, verbose = TRUE)

9 Calculate the mean background scale estimate per group as a ratio.


bg.scale.hk <‐ hk.clr@scaleSamps
bg.scale.h.hk <‐ mean(rowMeans(bg.scale.hk)[which(mts.meta$group == “Healthy”)])
bg.scale.bv.hk <‐ mean(rowMeans(bg.scale.hk)[which(mts.meta$group == “BV”)])
bg.scale.h.hk / bg.scale.bv.hk

  For this example, the ratio is 1:1.141893, so we can infer that the difference between groups is approximately 14%. We will use this ratio in our informed scale model to account for the scale difference between healthy and BV groups.

#### Differential expression analysis with an informed scale model

10 Set the seed value once more and use a built‐in ALDEx2 function to generate a matrix of scale uncertainty values to be used in the model. These values will be drawn from a log‐normal distribution with a standard deviation of 0.5 and will differ between groups by (on average) 14%.


set.seed(2025)
scale.f <‐ aldex.makeScaleMatrix(gamma = 0.5, mu = c(1, 1.14), conditions = mts.meta$group, mc.samples = 128)

11 Run the centered log‐ratio transformation on the full functional dataset, specifying a custom matrix of scale values to be used during the modeling of system scale.


scale.f.clr <‐ aldex.clr(reads = mts.ko, conds = mts.meta$group, mc.samples = 128, denom = “all”, gamma = scale.f, verbose = TRUE)

12 For each feature, calculate the median dispersion of log‐ratio abundances within groups and the median difference in log‐ratio abundances between groups. This is performed for all 128 Monte Carlo instances, and a median of medians is returned.


scale.f.clr.e <‐ aldex.effect(clr = scale.f.clr, verbose = TRUE, include.sample.summary = TRUE)

13 Conduct parametric and nonparametric tests to identify features for which the mean difference in the log‐ratio abundance between groups is significantly different, adjusting for multiple testing using the Benjamini‐Hochberg correction.


scale.f.clr.t <‐ aldex.ttest(clr = scale.f.clr, verbose = TRUE)

#### Visualize differential expression output

**Figure 2:**
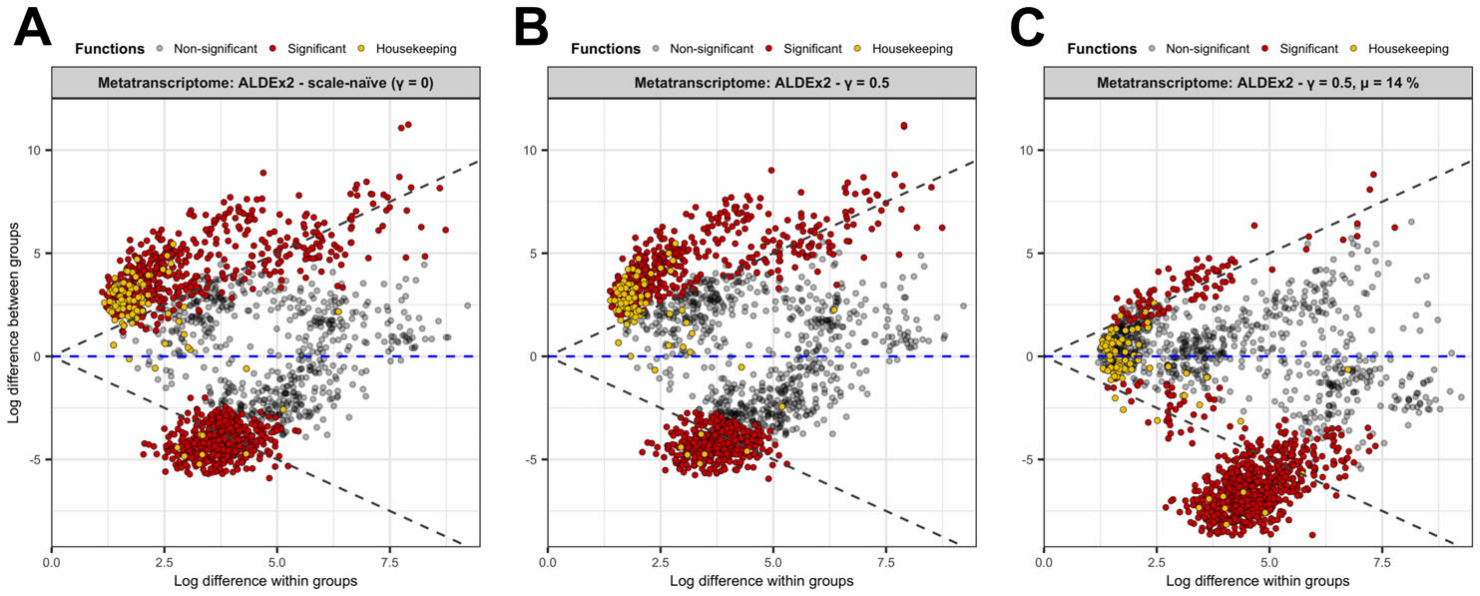
Implementing a full informed scale model corrects data asymmetry in problematic datasets. Count data from the vaginal metatranscriptome dataset were aggregated by KEGG orthology term prior to log‐ratio transformation using three different approaches: (**A**) a scale‐naïve analysis; (**B**) a simple scale model using gamma = 0.5; (**C**) a full scale model using gamma = 0.5 and a difference between conditions of 14%. Data are represented as effect plots. Black points represent nonsignificant functions; red points represent differentially expressed functions; yellow points represent housekeeping functions. Grey lines indicate equivalent difference within groups and difference between groups. Blue line indicates no difference between groups.

14 Visualize the output of ALDEx2 by creating an effect (difference in log‐ratio abundances between groups vs. dispersion of log‐ratio) for this analysis. ALDEx2 has a built‐in function for creating basic versions of this plot (not shown), but we also show the same data plotted using the ggplot2 R package in Figure 2 (see GitHub for ggplot2 plotting code).


scale.f.clr.all <‐ cbind(scale.f.clr.e, scale.f.clr.t)
aldex.plot(scale.f.clr.all, type = “MW”)

## Basic Protocol 3 VISUALIZING ALDEx2 OUTPUTS USING A COMPOSITIONAL APPROACH: PRINCIPAL COMPONENT ANALYSIS

There are a multitude of methods for visualizing and summarizing HTS data. Principal co‐ordinates analysis (PCoA), metric/non‐metric multidimensional scaling (MDS/nMDS), uniform manifold approximation and projection (UMAP), and *t*‐distributed stochastic neighbor embedding (*t*‐SNE) are frequently used methods in the study of microbiomes. Our preferred approach is to conduct principal component analysis (PCA) using the centered log‐ratio transformed abundances output by ALDEx2 and the prcomp() function built into R. This equates to calculating the Aitchison distance (i.e., the Euclidean distance between samples following CLR transformation), a truly linear distance metric that is sub‐compositionally coherent and robust to subsetting or aggregation of the underlying data (Aitchison, 1986; Gloor et al., 2017). In this protocol, we outline how to perform an exploratory analysis of HTS data using the output of ALDEx2 to produce a compositional biplot.

### Materials

Combined ALDEx2 outputs of your data from aldex.clr(), aldex.effect(), and aldex.ttest(). Here we use pre‐computed data from Dos Santos et al. (2024) (see data/mts_scaleFull.Rda from the current study's GitHub repository). This is equivalent to the combined dataframe produced above (see Basic Protocol 2, step 14).
Computer with R and RStudio installed and >8 GB RAM. ALDEx2 and its dependencies must be installed from Bioconductor (pre‐computed ALDEx2 outputs are provided for replication of this protocol as an alternative for those who do not meet this requirement). For this protocol, R (v4.5.1) and RStudio (2024.12.1, build 563) were used, along with the following R packages: BiocManager (v1.30.26), ALDEx2 (v1.41), CoDaSeq (v0.99.7), dplyr (v1.1.4), ggnewscale (v0.5.2), and ggplot2 (v3.5.2).

#### Environment setup

1 Follow the previous protocols (see Basic Protocol 1, steps 1‐6, or Basic Protocol 2, steps 1‐14) to produce a combined ALDEx2 output from the aldex.clr(), aldex.effect(), and aldex.ttest() functions. If replicating this example, load in the pre‐computed combined ALDEx2 output for the KO‐aggregated vaginal metatranscriptomes in Dos Santos et al. (2024).


url.scaleFull <‐ “https://github.com/scottdossantos/currprotSDS/raw/refs/heads/main/data/mts_scaleFull.Rda”
load(url(url.scaleFull))

##### Perform PCA

2 Extract the centered log‐ratio abundance values for all features and samples from the ALDEx2 output (these columns all start with rab.sample.). Remove rab.sample. from the column names.


scale.f.lr <‐ scale.f.clr.all[, grep(“rab.sample.”, colnames(scale.f.clr.all))]
colnames(scale.f.lr) <‐ gsub(“rab\\.sample\\.”, “”, colnames(scale.f.lr))

3 Perform the principal component analysis using the prcomp() function.


pca.f <‐ prcomp(t(scale.f.lr))

  Input must be transposed, as the function expects samples in rows and features in columns. Note that performing PCA on CLR‐transformed data is equivalent to building an ordination based on Aitchison distances (Euclidean distances between samples following CLR‐transformation).

###### Visualize biplot: base R

4 To plot a very basic compositional biplot, use the built in plot() function.


biplot(pca.f, cex = c(0.55, 0.25), col = c(“blue”, rgb(0,0,0,0.1)))

###### Visualize biplot: CoDaSeq

5 For more customization, the CoDaSeq package has a more advanced plotting function specifically for visualizing HTS data. As an example, we will plot a compositional biplot that highlights the KEGG pathways that several differentially expressed KO terms belong to, with all other KO terms plotted as transparent points. Start by making a list that contains the indices of the samples in the grouping vector.


ind.grp <‐ list(Healthy = which(mts.meta$group == “Healthy”), BV = which (mts.meta$group == “BV”))

  To color samples or features, the codaSeq.PCAplot() function requires a list that contains the groups of data you want to highlight (e.g., treatment groups, disease vs. control for sample labels; taxa or pathway membership for features) and the column/row indices of each sample or feature in the input data.
6 Make a second list that contains the indices of the KO terms to highlight in the KO to pathway lookup table, grouped by pathway.


pathways <‐ c(“Aminoacyl‐tRNA biosynthesis”,“Bacterial chemotaxis”,“Butanoate metabolism”,
“CAMP resistance”,“Exopolysaccharide biosynthesis”,“Flagellar assembly”,
“Porphyrin metabolism”,“Ribosome”,“Starch and sucrose metabolism”,
“Two‐component system”)
ind.load <‐ list()
for(i in pathways){ind.load[[i]] <‐ which(mts.func$pathway == i)}

7 Add all remaining KO terms to a list element called ‘Other’. These will be displayed as transparent grey points.


ind.load[[“Other”]] <‐ setdiff(1:nrow(scale.f.lr), unlist(ind.load))

8 Make two vectors of R‐recognized colors, one for groups and another for pathways.


cols.group <‐ c(“dodgerblue2”, “orange2”)
cols.load <‐ c(“skyblue1”,“red3”,“gold2”,“chocolate4”,“greenyellow”,“purple3”,“olivedrab”,“black”,“maroon”,“cyan3”,rgb(0,0,0,0.075))

9 Plot the compositional biplot with codaSeq.PCAplot(), adding density plots for the highlighted pathways, a title, and a legend for the pathways. Samples will be colored by group and KO terms will be colored by pathway.


codaSeq.PCAplot(pca.f, plot.groups = T, plot.loadings = T, plot.density = “groups”,
PC = c(1,2), grp = ind.grp, grp.col = cols.group, grp.cex = 0.75,
load.grp = ind.load, load.col = cols.load, load.sym = 19,
load.cex = 0.6, plot.legend = “loadings”, leg.position = “bottomright”,
leg.columns = 2, leg.cex = 0.625,
title = “Vaginal metatranscriptome: PCA plot (\u03b3 = 0.5, \u03bc = 14 %)”)

###### Visualize biplot: ggplot2

10 Alternatively, if complete control over the plots is desired, extract the scaled coordinates for PC1 and PC2 from the PCA object for both the samples and KO terms. These will be used as input data for ggplot2.


load.f.samp <‐ data.frame(pca.f$x)[,1:2]
load.f.feat <‐ codaSeq.PCAvalues(pca.f)[,1:2]

  The data in the PCA object giving the sample scores (pca.f$x) corresponds to the sample coordinates on the PCA plot; however the loadings (pca.f$rotation) do not correspond to the coordinates of the features. This CoDaSeq function scales the loadings to the PCA scores, allowing you to plot the same biplot however you like.
11 Add pathways to the dataframe containing the feature coordinates.


for(i in 1:length(ind.load)){load.f.feat[ind.load[[i]], “path”] <‐ names(ind.load)[i]}

12 Add a column for setting the outline of the data point (either transparent grey or solid black) based on whether the KO should be highlighted or not.


load.f.feat$col <‐ case_when(load.f.feat$path == “Other” ∼ rgb(0,0,0,0.05), .default = “black”)

13 Convert the pathway column to a factor and re‐order the levels to ensure that KO terms belonging to the ‘Other’ pathway are plotted last (as they are transparent).


lvs <‐ levels(factor(load.f.feat$path))
load.f.feat$path <‐ factor(load.f.feat$path, levels = c(lvs[1:6], lvs[8:11], lvs[7]))

14 Add group information and a plot title to the dataframe containing the sample coordinates.


for(i in 1:length(ind.grp)){load.f.samp[ind.grp[[i]], “group”] <‐ names(ind.grp)[i]}
load.f.samp$title <‐ “Vaginal metatranscriptome: PCA plot (\u03b3 = 0.5, \u03bc = 14 %)”

15 Make a vector to (1) ensure the order of the group colors and (2) change the legend symbol for the samples (text) from ‘a’ to a more appropriate symbol (in this case, ‘h’ and ‘v’).


grp_labels<‐ c(Healthy = “h”, BV = “v”)

16 Plot the compositional biplot using ggplot2.


ggplot(data = load.f.samp, aes(x = PC1, y = PC2))+
geom_hline(yintercept = 0, colour = “grey50”, linewidth = 0.5, linetype = 2)+
geom_vline(xintercept = 0, colour = “grey50”, linewidth = 0.5, linetype = 2)+
geom_text(aes(colour = group), label = rownames(load.f.samp))+
scale_colour_manual(name = “Group”, values = cols.group, breaks = names (grp_labels),
guide = guide_legend(override.aes = list(label = grp_labels)))+
new_scale_colour()+
geom_point(data = load.f.feat, aes(fill = path, colour = col),
stroke = 0.25, shape = 21, size = 2)+
scale_fill_manual(name = “Pathway”, values = cols.load,)+
scale_colour_manual(name = “Pathway”, values = c(rgb(0,0,0,0.05), “black”))+
xlab(“PC1: 49.4 % variance explained”)+
ylab(“PC2: 11.0 % variance explained”)+
guides(colour = “none”)+
theme_bw()+
facet_wrap(∼title)+
theme(strip.text = element_text(size = 9, face = “bold”),
panel.grid.major = element_blank())

## COMMENTARY

### Critical Parameters

The most important parameter for these differential expression analyses is the value passed to the gamma argument of aldex.clr(), which corresponds to the standard deviation of the log‐normal distribution from which the scale estimates are drawn. Therefore, gamma essentially represents the degree of scale uncertainty that ALDEx2 will model. Consider the following example of volcano plots showing the ALDEx2 analysis of the yeast transcriptome dataset (Fig. 1). When running a scale‐naïve analysis (i.e., the model does not accept any uncertainty when estimating system scale), 4169/5891 genes (70.7%) are called as significantly different between wild‐type and *Snf2* knockout cells (Fig. 1A). Only 189 of these are considered truly different when implementing a secondary log_2_ fold‐change threshold of 1.4 in either direction (the approximate midpoint of the high and low fold‐change thresholds proposed by the original authors; Gierliński et al., 2015; Schurch et al., 2016). By implementing a simple scale model and setting gamma to 0.5 (i.e., the log‐normal distribution that the scale estimates are drawn from has a standard deviation of 0.5), only 175 genes are called as significantly different (Fig. 1B), achieving the same result as a fold‐change cutoff without the need for arbitrarily deciding what that threshold should be and unintentionally increasing the FDR in the process. Figure 1C,D shows effect plots for the corresponding analyses of the yeast dataset. When accounting for scale uncertainty, many features with a high within‐group dispersion are no longer reported as differentially expressed.

In some cases, a simple scale model with a single gamma value is insufficient for proper analysis of a dataset. This issue is clearly demonstrated in Figure 2A, which shows an effect plot (log dispersion vs. log difference) of the vaginal metatranscriptome dataset in a scale‐naïve ALDEx2 analysis. Positive log‐difference values indicate a higher expression in samples from healthy patients while negative values indicate higher expression in samples from BV patients. Points in yellow are housekeeping functions that are largely universal and are expected to be invariant between groups (e.g., glycolysis/tRNA biosynthesis/ribosomal functions) (Joshi et al., 2022). However, the expression of many of these genes is also deemed to be significantly different (red points; *n* = 1089), pointing to a problem during the normalization. Adding a modest amount of scale uncertainty with a simple scale model does little to address this problem (Fig. 2B). The dispersion of all data points has increased slightly, and a few likely false‐positive findings are now no longer significantly different (*n* = 934; compare points just inside the grey lines of equivalence for dispersion and difference in Fig. 2A,B). However, the bulk of these housekeeping functions are still estimated to be significantly overexpressed in healthy metatranscriptomes. These housekeeping functions are only correctly centered around the line of no difference when an informed scale model is applied, using a scale difference between groups of 14% (Fig. 2C). This value corresponds to the average background scale estimate for each group, as determined in Basic Protocol 2, steps 6‐9. When the full, informed model is used, the total number of differentially expressed genes drops to 875, eliminating over 200 false discoveries relative to the initial analysis. Comparing the scale‐naïve and informed scale models, one can see many false‐positive and false‐negative findings are present in the unscaled output when uncertainty around the system scale is not accounted for during normalization.

The above two examples beg the question how does one choose an appropriate gamma value? Unfortunately, there is no simple concrete answer. Larger gamma values will reduce the number of features called as significantly different, but increase the confidence that features identified as differential are truly so. Conversely, lower gamma values or scale‐naïve analyses will return a larger number of differential features but with the risk that a proportion of these features represent false‐positive findings. We have previously argued that there is no such thing as a “free lunch” when it comes to statistical analysis of ‐omics data (Gloor et al., 2025) and that this extends to tools beyond ALDEx2, which will always force the analyst into a trade‐off of prioritizing sensitivity or good FDR control but never both. These observations have been replicated by others in one of the most comprehensive benchmarking studies of over 15,000 samples from 35 studies (Konnaris et al., 2025). We have further demonstrated this in recent benchmark work comparing ALDEx2 to several widely used differential expression analysis tools such as DESeq2, edgeR, and limma (Dos Santos et al., 2025).

**Figure 3:**
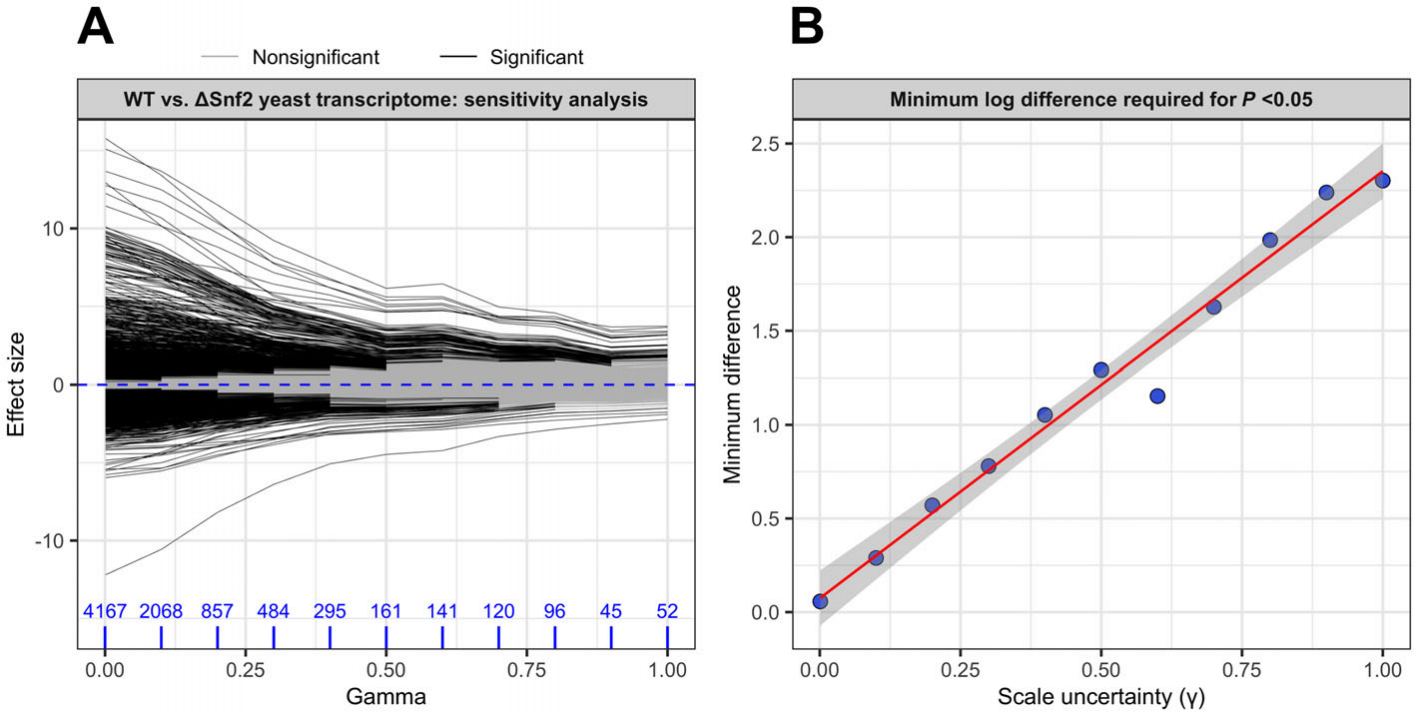
Sensitivity analysis informs the selection of an appropriate gamma value. Count data from the yeast transcriptome dataset were used for a sensitivity analysis in ALDEx2 with a range of gamma values from 0 to 1.0 in increments of 0.1. (**A**) Effect sizes at each gamma value are plotted for all features and colored by significance at each gamma value. Blue numbers indicate the number of differentially expressed features at each gamma value. (**B**) The minimum absolute log difference between wild‐type and *Snf2* knockout cells required for a statistically significant result is shown for each gamma value. The regression line represents a slope of 2.2.

ALDEx2 contains built‐in functions to allow researchers to identify features that are particularly sensitive to increasing values of gamma (i.e., those that were already on the margin of significance without accounting for even small amounts of scale uncertainty). The aldex.senAnalysis() function takes a vector of numeric values and repeats the differential expression analysis sequentially using each of the supplied values for the gamma argument. Likewise, the plotGamma() function creates a visualization showing the effect sizes of all features in the dataset at each specified gamma value, with significance at a given gamma value indicated by color (i.e., shows how increasing amounts of scale uncertainty affect the strength of findings in the differential expression analysis). An example output of the aldex.senAnalysis() function is shown in Figure 3A for the yeast transcriptome dataset. Adding even a minor amount of scale (0.1) reduces the number of significantly expressed genes between wild‐type and *Snf2* knockout cells by 50%. Many of these genes were likely only significant due to marginal significance or difference values (Fig. 1A). Accordingly, we recommend gamma values between 0.2 and 0.5, but ultimately the analyst has to choose the extent to which they will tolerate false discoveries.

To further inform the choice of gamma value, we can ask what is the smallest log difference between groups that will result in a significant result (*P* < 0.05) at a given value of gamma? Figure 3B shows a linear regression of the minimum log difference required for significance vs. increasing gamma values for the yeast transcriptome dataset. As the degree of scale uncertainty increases, so too does the log difference needed to identify a feature as significantly different between conditions. Notably, the slope of the regression line is approximately 2.5, meaning that analysts can expect a given gamma value to require a log difference between their biological conditions of interest roughly 2.5 times as large as the gamma value they passed to aldex.clr() during normalization. We have found that the relationship between gamma and the minimum log‐difference required for a significant result is relatively consistent, even when testing different types of ‐omics data, including bulk RNA‐seq, single‐cell RNA‐seq, and metatranscriptome data (Dos Santos et al., 2025). Accordingly, analysts should consider how much of a difference exists between their biological conditions of interest. If there is a large and evident difference between conditions (e.g., immediately obvious when visualizing beta diversity), a larger gamma value will be appropriate. In the opposite case, where there is not much difference between conditions, a small value will suffice. In either case, modeling scale uncertainty with ALDEx2 will offer excellent control of the false‐discovery rate at any given gamma value. We recommend at minimum that users employ a gamma value of 0.2, equating to a minimum log_2_ fold‐change of 0.5 (i.e., a ∼1.4‐fold difference between groups) for any significant result. This gives an excellent control of the FDR while maintaining sensitivity and removes the need for any dual‐cutoff approach.

In addition to nonparametric significance tests, ALDEx2 can also construct generalized linear models (GLMs) via the aldex.glm() and glm.effect() functions. Scale models can be used in these functions in the exact same manner as outlined in both protocols above: through the gamma parameter when calling aldex.clr(). The successor to ALDEx2, the aptly named ALDEx3, is currently in development and is based on linear models. ALDEx3 permits modeling of scale uncertainty in the same manner as its predecessor, but it is much faster and is no longer bound by memory constraints when analyzing large datasets. For researchers looking to employ linear models in their work, ALDEx2's GLM function remains an option until ALDEx3 is formally released.

### Troubleshooting

For troubleshooting guidelines, see Table 1.

**Table 1:** Troubleshooting Guide for Running ALDEx2 on Any ‐Omics Dataset

Problem	Possible cause(s)	Solution
Memory‐related errors while running ALDEx2 in R	Machine used for running ALDEx2 has insufficient RAM	Lower the number of Monte Carlo instances used to model the underlying variation in count data (controlled by the mc.samples argument in the aldex.clr() function)
		Alternatively, run ALDEx2 on a server with sufficient RAM
ALDEx2 reports mismatch between number of samples and conditions vector	Count data table is in the opposite orientation (i.e., samples in rows and features in columns)	Confirm that count data has samples in columns and features in rows
		Check that the count data table has been imported into R correctly and has the expected number of rows and columns (e.g., feature names are given in an actual column in the dataframe rather than set to the row names attribute)
ALDEx2 reports no significant differences between groups	Gamma value is too high	Perform a sensitivity analysis using a range of gamma values from low to high to visualize the effect of adding scale uncertainty on ALDEx2 outputs
	Incorrect grouping vector supplied	Confirm that grouping vector is correct and in the same order as the samples in the count data

### Understanding Results

The core ALDEx2 functions, aldex.effect() and aldex.ttest() return dataframes that can be combined for plotting. Below, we explain what information is contained within the columns of these dataframes.

**rab.all**: The median log‐ratio abundance values for each feature across all samples, itself expressed as a median calculated across all Monte Carlo instances. These are always relative to the geometric mean value of the samples.

**rab.win.A**: The median log‐ratio abundance values for each feature across all samples in group A, itself expressed as a median calculated across all Monte Carlo instances.

**rab.win.B**: The median log‐ratio abundance values for each feature across all samples in group B, itself expressed as a median calculated across all Monte Carlo instances.

**rab.sample.X**: The median log‐ratio abundance value for a given feature in sample X across all Monte Carlo instances.

**diff.btw**: The log_2_ fold‐change between conditions, calculated as in Fernandes et al. (2014).

**diff.win**: The pooled dispersion of the feature, calculated as in Fernandes et al. (2014). This is analogous to a pooled standard deviation.

**effect**: A standardized measure of effect sizes given by dividing diff.btw by diff.win for a given feature, calculated as a median across all Monte Carlo instances.

**overlap**: The proportion of effect sizes that overlap zero (i.e., the percentage of Dirichlet instances in which the effect size was in the opposite direction). An overlap of 0.5 indicates that the two distributions share the same midpoint.

**we.ep**: The posterior predictive *P* value from Welch's *t*‐test for each feature.

**we.eBH**: The Benjamini‐Hochberg‐corrected posterior predictive *P* value from Welch's *t*‐test for each feature.

**wi.ep**: The posterior predictive *P* value from the Wilcoxon rank sum test for each feature.

**wi.eBH**: The Benjamini‐Hochberg‐corrected posterior predictive *P* value from the Wilcoxon rank sum test for each feature.

### Time Considerations

The amount of time required to run the main functions of the ALDEx2 package for differential expression analysis will vary depending on the number of samples and number of features in the read count table as well as the number of Monte Carlo instances specified in the call to the aldex.clr()function. Table 2 shows the time taken to run the three core modular functions of the ALDEx2 package for transformation, effect size calculation, and significance testing in RStudio (all run on a 2023 MacBook Pro with 16 GB RAM and an Apple M2 Pro processor). ALDEx2 does support multicore processing for the aldex.clr() and aldex.effect() functions, which is implemented via the BiocParallel package. However, due to the amount of overhead required by R to set up parallel processes, this does not offer much of an improvement in speed, particularly when calculating effect sizes (Table 2). Note that all simulations were performed while no applications other than RStudio were running.

**Table 2:** Time for computation of log‐ratio abundances, effect sizes, and posterior predictive *P* values*^a^* *^,^* *^b^*

Dataset	Size (features × samples)	Time (seconds ± s.d.)	
		Single core	Multi‐core
Yeast transcriptome	5891 × 86	54 (1.1)	52 (1.6)
Vaginal metatranscriptome	1658 × 42	10 (0.2)	10 (1.2)
PD1 immunotherapy	20,449 × 109	402 (12.0)	460 (43.5)

^*a*^ Times determined when running ALDEx2 on the yeast transcriptome and vaginal metatranscriptome datasets as well as a large RNA‐seq dataset from cancer cells pre‐ and post‐exposure to the PD1 antagonist nivolumab (data taken from Li et al., 2022).

^*b*^ Times represent a mean of five iterations rounded to the nearest integer, with standard deviations in parentheses. Computation was performed on a 2023 MacBook Pro with an Apple M2 Pro processor and 16 GB of RAM, with no applications running other than RStudio.

### Author Contributions

**Scott Dos Santos**: Conceptualization; formal analysis; methodology; software; visualization; writing—original draft; writing—review and editing. **Gregory Gloor**: Conceptualization; formal analysis; methodology; software; supervision; visualization; writing—original draft; writing—review and editing.

### Conflict of Interest

GBG is an author and current maintainer of ALDEx2.

## Data Availability

All code required for replicating these analyses and producing the figures shown in the article can be found on this study's GitHub repository at https://github.com/scottdossantos/currprotSDS, along with data tables containing read counts per sample and metadata.
